# Severe lactic acidosis and acute renal failure following ingestion of metformin and kerosene oil: a case report

**DOI:** 10.1186/1752-1947-6-18

**Published:** 2012-01-17

**Authors:** Amila Rathnapala, Thushara Matthias, Saroj Jayasinghe

**Affiliations:** 1 University Medical Unit, National Hospital, Colombo, Sri Lanka; 2 Department of Clinical Medicine, Faculty of Medicine, University of Colombo, Colombo, Sri Lanka

## Abstract

**Introduction:**

Kerosene is a freely accessible hydrocarbon used in Sri Lankan (and other Asian) households for cooking and for lighting lamps. Kerosene poisoning is rarely reported among adults and its toxicological effects are not well known. Metformin is a commonly used oral hypoglycemic drug and its overdose leads primarily to lactic acidosis. Combined poisoning of metformin and kerosene and their interactions have not been reported.

**Case presentation:**

An 18-year-old, previously healthy, unmarried Sinhalese woman was referred following ingestion of 17.5 g of metformin and approximately 200 mL of kerosene oil in a suicide attempt. She had vomiting, burning epigastric pain, and a hypoglycemic seizure (capillary blood glucose of 42 mg/dL). Subsequently, she developed severe lactic acidosis followed by acute renal insufficiency, was treated with sodium bicarbonate, and underwent intermittent hemodialysis with bicarbonate. She recovered completely.

**Conclusions:**

This report proposes possible interactions that occur between metformin and kerosene that augment toxicity when the two are ingested together. It also stresses the importance of early treatment with intermittent hemodialysis in severe lactic acidosis with maintenance of blood glucose.

## Introduction

Metformin is one of the commonest drugs used as a treatment of type 2 diabetes mellitus and in insulin resistance states such as polycystic ovarian syndrome. Lactic acidosis is a recognized complication of pharmacological doses of the drug, mostly in those with chronic renal failure, and has a frequency of 0.01 to 0.08 per 1000 patient-years [1]. Overdose with metformin leads to severe lactic acidosis and fatalities. The pathogenesis is not well known.

Kerosene is a freely accessible hydrocarbon used in Sri Lankan (and other Asian) households for cooking and for lighting lamps. It is also used as a solvent of many substances, including pesticides. Kerosene poisoning is rarely reported among adults, although epidemiological studies indicate high rates of hydrocarbon poisoning. Its effect on kidneys is not well documented and is likely to lead to acute tubular necrosis [2].

There is a debate in the literature about lactic acidosis caused by pharmacological doses of metformin. A study found that metformin, despite concerns to the contrary, is not associated with an increased risk for lactic acidosis compared with other anti-hyperglycemic treatments, whereas others have reported a low incidence of lactic acidosis in those on metformin [3]. However, metformin is well known to cause lactic acidosis after an overdose [4]. We report a case of metformin and kerosene toxicity with hypoglycemia, severe acidosis, and renal failure and suggest the possibility of pharmacokinetic and metabolic interactions to explain acidosis observed in our patient.

## Case presentation

An 18-year-old, previously healthy, unmarried Sinhalese woman from a nearby city (20 km away from Colombo) was referred to a tertiary care hospital in Colombo for treatment of metabolic acidosis after an overdose of 35 tablets of metformin (17.5 g) and a quarter bottle (nearly 200 mL) of kerosene oil. She was admitted to a local hospital a few hours after ingestion and was transferred to a teaching hospital in Ragama, where she developed a generalized tonic clonic seizure that lasted five minutes. When she was admitted to the teaching hospital, her capillary blood sugar level had been 42 mg/dL and was treated with a bolus intravenous dextrose and 10% dextrose infusion. Her arterial blood gas (ABG) revealed a severe metabolic acidosis (Table 1). She was treated with two doses of 75 mL of 8.4% sodium bicarbonate (NaHCO_3_) (75 mmol) but had persisting acidosis three hours later. At that stage, she was given 50 mL of NaHCO_3 _(50 mmol), 500 mg ampicillin, and 50 mg ranitidine (all intravenously) and was transferred to a Tertiary care hospital, Colombo (THC) for hemodialysis.

**Table 1:** Arterial blood gas reports

	Duration from the time of poisoning						
	11 hours	15 hours	24 hours	28 hours	48 hours	51 hours	67 hours
pH	7.081	7.167		7.1	7.386	7.42	7.43
Bicarbonate, mmol/L	4	4		< 3	16.5	23.4	21.9
Base excess, mmol/L	-25.9	24.4		NA	-8.6	-1.1	-2.4
Lactate, mg/dL			59.96				

pH and serum bicarbonate levels progressively decreased until 28 hours after self-poisoning and improved gradually with recurrent hemodialysis. NA, not applicable.

At THC, she complained of burning epigastric pain and shortness of breath. She also had several episodes of vomiting. Her peripheries were warm. She had Kussmaul breathing and a respiratory rate of 44/minute, a pulse rate of 116/minute, and a blood pressure of 90/60 mm Hg. Except for the mild epigastric tenderness, the results of the rest of a physical examination were normal. Her oxygen saturation was 92% with 5 L/minute oxygen via a face mask. The ABG at 15 hours after ingestion showed continuing acidosis with a high anion gap (Table 1). Her serum lactate level was elevated at 59.96 mg/dL.

**Figure 1:**
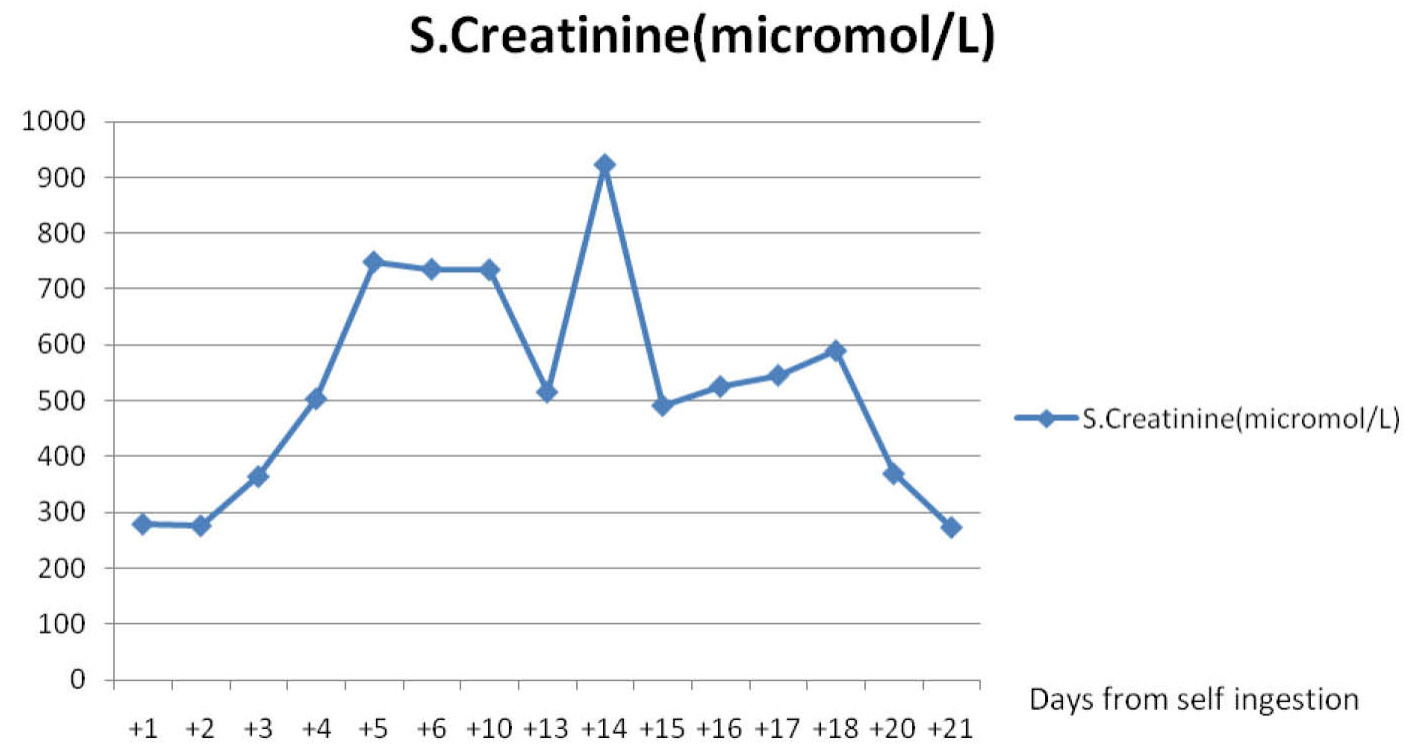
**Change of serum creatinine level over time**.

Eighteen hours after ingestion, she was started on intermittent hemodialysis with bicarbonate buffer (which contains both solutions A and B) (Additional file 1). The initial dialysis was continued only for two and a half hours with a blood flow rate of 140 mL/minute and a dialysate flow rate of 500 mL/minute. However, she continued to be acidotic four hours after the dialysis (that is, 28 hours after ingestion). She was treated again with 8.4% NaHCO_3 _(100 mL), and dialysis was repeated on the following day. The ABG after the second dialysis (that is, 48 hours following ingestion) showed compensated metabolic acidosis (Table 1). The serum creatinine level was 279 μmol/L on admission and started to increase rapidly two days after admission (Figure 1) and was accompanied by a declining urine output. Subsequently, she was dialyzed on days 3, 10, and 14. All of the subsequent dialyses were continued for 4 hours with a blood flow rate of 200 mL/minute and a dialysate flow rate of 500 mL/minute. Her urine output and renal functions improved gradually, and after three weeks she was discharged with normalized renal functions.

## Discussion

This case highlights some of the interesting effects of metformin overdose. Our patient developed lactic acidosis, which is well recognized with overdose and carries a mortality of 50% [5]. In the literature, there is a debate about lactic acidosis caused by pharmacological doses of metformin. Salpeter and colleagues [6] reviewed all studies of metformin treatment from 1966 to August 2005 and found no cases of fatal or nonfatal lactic acidosis. Also, there was no disparity in lactate levels between metformin and placebo or other treatment groups. The authors concluded that there is no evidence that metformin is associated with an increased risk of increased lactate levels or lactic acidosis. The precise pathophysiology of lactic acidosis in metformin overdose is not well known. It could be that the drug decreases pyruvate dehydrogenase (a mitochondrial reducing enzyme) activity and enhances anaerobic metabolism, which leads to increased metabolism of pyruvate to lactate [7]. Another hypothesis is that metformin accumulates in the intestine and increases the production of lactate, which lowers the pH within the liver and decreases lactate metabolism by suppressing pyruvate carboxylase [8]. A prospective observational study conducted by Arroyo and colleagues [9] from 2006 to 2010 among 29 patients on pharmacological doses of metformin revealed that acute kidney injury is associated with an episode of volume depletion due to gastrointestinal losses. Similarly, hypotension due to vomiting at the time of admission in our patient might have contributed to the severity of metabolic acidosis and acute renal failure.

There are few reports of kerosene toxicity and its effects on metabolic pathways. Kerosene inhibits cellular respiration processes of the liver and kidney *in vitro*. This leads to an increase in concentration of lactate and pyruvate in the blood and liver, a decrease in glucose concentration in the blood, and a reduction of glycogen content in the liver and in the skeletal muscle and is associated with a concomitant increase of lactate dehydrogenase activity in the liver [10]. Kerosene ingestion causes renal tubular necrosis, which could have led to impaired elimination of metformin, as it is eliminated primarily via the kidneys with negligible metabolism [11].

We also hypothesize a synergistic effect of kerosene and metformin on the cellular respiration to explain the prolonged hypoglycemia and acidosis in our patient. It is well known that metformin leads to hypoglycemia due to decreased hepatic glucose output [12] and increased glucose utilization by muscles and liver (which is insulin-mediated) [13]. The inhibition of respiration by kerosene, described in the previous paragraph, and the effects of metformin at the cellular level could act synergistically to result in prolonged hypoglycemia and increased lactic acid production.

The mainstay of therapy involves repairing acid-base balance, removing causes of lactic acidosis, and supportive therapy. Hemodialysis with bicarbonate buffer has been used successfully in the treatment of lactic acidosis in metformin use [14]. Hemodialysis not only corrects the acidosis but also efficiently removes metformin from plasma, preventing further lactate overproduction, and removes lactate [15].

This case report reiterates the fact that lactic acidosis can be corrected by the above means as the patient made a full recovery from acute renal failure. This case report adds to the series of case reports that have used hemodialysis as a treatment modality for metformin-induced lactic acidosis, although hemofiltration is considered to be the intervention of choice [16]. Bicarbonate buffer too has been used to combat the severe acidosis from metformin overdose [17].

## Conclusions

This case report highlights the relative lack of knowledge in relation to acute human toxicity from two commonly used agents. The paper proposes possible interactions that occur between metformin and kerosene that augment toxicity when the two are ingested together. It also stresses the importance of early treatment with intermittent hemodialysis in severe lactic acidosis with maintenance of blood glucose.

## Abbreviations

ABG: arterial blood gas; NaHCO_3_: sodium bicarbonate; THC: Tertiary Care Hospital, Colombo.

## Consent

Written informed consent was obtained from the patient for publication of this case report and any accompanying images. A copy of the written consent is available for review by the Editor-in-Chief of this journal.

## Competing interests

The authors declare that they have no competing interests.

## Authors' contributions

AR and TM were involved in analysis and interpretation and in drafting the manuscript. SJ supervised and revised it critically and gave intellectual inputs to the discussion. All authors read and approved the final manuscript.

## Supplementary Material

Additional file 1 **Constituents of bicarbonate buffer**. The bicarbonate buffer comes in two solutions, named A and B. Click here for file
