# Supplementary material for: Severe lactic acidosis and acute renal failure following ingestion of metformin and kerosene oil: a case report
Source: J Med Case Rep. 2012 Jan 17;6:18. doi: 10.1186/1752-1947-6-18 (PMC3292833; doi:10.1186/1752-1947-6-18)
Supplement: Additional file 1 — Constituents of bicarbonate buffer. The bicarbonate buffer comes in two solutions, named A and B. [file 1752-1947-6-18-S1.PDF]

Additional file 1

Constituents of bicarbonate buffer

The bicarbonate buffer comes in two solutions, named A and B.

Solution **A**	Solution **B**
Na 75 mmol/l<br><br>K 2.0mmol/l<br><br>Ca 1.8<br><br>Mg 0.5<br><br>Cl 81.6<br><br>CH_3COO^- 4.0<br><br>HCO_3^- 35<br><br>Osmolality 290.9 mOsm/l	NaCl 30.5 g/l<br><br>$NaHCO_3$ 66.0g/l
